# Effect of sintering time on the marginal and internal fit of monolithic zirconia crowns containing 3–4 mol% Y_2_O_3_

**DOI:** 10.1186/s12903-022-02563-x

**Published:** 2022-11-15

**Authors:** Yunus Emre Ozden, Mustafa Baris Guncu, Guliz Aktas, Senay Canay

**Affiliations:** 1 Department of Prosthodontics, Faculty of Dentistry, Yeditepe University, İstanbul, Turkey; 2 Department of Prosthodontics, Faculty of Dentistry, Hacettepe University, Ankara, Turkey

**Keywords:** Monolithic, Zirconia, Sintering, Marginal, Fit

## Abstract

**Background:**

Short-term sintering may offer advantages including saving time and energy but there is limited evidence on the effect that altering sintering time has on the accuracy of monolithic zirconia crowns. The purpose of this in vitro study was to investigate the effect of shortened sintering time on the marginal and internal fit of 3Y-TZP and 4Y-TZP monolithic crowns.

**Methods:**

Sixty monolithic zirconia crowns were fabricated for the maxillary first molar tooth on the prefabricated implant abutment. Groups were created according to the material composition: 3Y-TZP Generation 1, 3Y-TZP Generation 2 and 4Y-TZP. Two different sintering protocols were performed: same final sintering temperature (1500 °C) and various rates of heating (10 °C/min and 40 °C/min), cooling down speed (− 10 °C/min and − 40 °C/min), holding time (45 and 120 minutes), and total sintering time (approximately 2 and 7 hours, respectively). The marginal and internal fit of the crowns were determined using the silicone replica technique. Comparisons between groups were analyzed using two-way ANOVA. Pairwise multiple comparisons were performed using t-test (*p* < 0.05).

**Results:**

The mean marginal gap values of 4Y-TZP zirconia revealed statistically significant increase for the short-term sintering protocol (*p* < 0.0001), while no difference was observed between the sintering protocols for the mean marginal gap values of 3Y-TZP groups. Although all groups showed clinically acceptable gap values, altering the sintering time had an effect on marginal fit of the crowns manufactured from 4Y-TZP zirconia.

**Conclusions:**

Shortening the sintering time may lead to differences within clinically acceptable limits. The manufacturer’s recommendations according to material composition should be implemented with care.

## Background

Among the all-ceramic restorations, the zirconia structures show statistically higher values of biaxial flexural strength and indentation fracture toughness [1]. Stabilizing oxides added to the crystal lattice of zirconium are of great importance in these superior mechanical properties. Yttrium oxide (Y_2_O_3_ or yttria) is one of the most commonly used stabilizers for zirconia compositions [2]. Until 2012, only high-strength 3Y-TZP Generation 1 (approximately 5.2 wt%/ 3 mol%/ alumina wt ≤0.5%) was used for fabricating restorations from single crowns to multi-unit bridges. To achieve a higher transmittance of light with good long-term stability and high strength, the number and grain size of the alumina (Al_2_O_3_) grains were reduced [3]; Y-TZP Generation 2 (approximately 5.2 wt%/ 3 mol%/ alumina wt ≤0.05%) was introduced.3 While this attempt maintained the zirconia’s mechanical properties, the optical characteristics were still insufficient [4]. The translucent dental zirconias involved increasing the content of yttria [2]. Due to the increased yttria content, cubic phase occurs alongside metastable tetragonal phase. The quantity of the cubic phase increases around 25% in 4Y- TZP. The grains in 4Y-TZP are larger than in 3Y-TZP, resulting in fewer grain boundaries, less birefringence and scattering of light [5].

With the introduction of modified translucent zirconia materials, the use of fully anatomic zirconia crowns and fixed partial dentures in the posterior region has become a new restorative option [6]. Monolithic zirconia offers two advantages: minimal tooth reduction since there is no need for space clearance for the veneering material [7] and reduced cost and time for the production compared with porcelain-fused-to-zirconia [8]. Although the monolithic approach is not new, monolithic Y-TZP posterior restorations have a significant advantage due to their high fracture resistance [9, 10].

Zirconia restorations are milled from premanufactured blocks using the CAD/CAM technology. Commercially, these blocks are available in two process stages: presintered and fully sintered [11]. Milling from presintered blocks is easier, faster, and causes lesser wear on the machining tools. Due to these advantages, presintered blocks are popular for the fabrication of zirconia structures in the world market. When presintered blocks are used, the material must be sintered at a high temperature in order to obtain sufficient strength after the milling process. During the sintering process, the material shrinks making the framework denser and stronger [12]. Moreover, sintering temperature and duration affects the microstructure of the Y-TZP. The mean grain size in 3Y-TZP increases with the sintering temperature and time [13, 14]. The process should be performed at sufficiently low temperatures to avoid the occurrence of a dual cubic–tetragonal microstructure but also at sufficiently high temperatures to obtain fully dense materials [14].

Short-term sintering may offer several advantages including improved productivity as well as saving time and energy. A few studies have been conducted to examine the effect of sintering time on the mechanical properties of 3Y-TZP [15–18]. The previous studies have been controversial. While Juntavee et al. [18] suggested that prolonging the sintered-holding time lead to enhancing the flexural strength of the translucent monolithic zirconia, Ebeid et al. [15] reported that biaxial flexural strength is not affected by changes in the sintering times. Moreover, Ersoy et al. [16] advised the combination of high temperature and short sintering time to increase the flexural strength of zirconia.

While one of the most important factors affecting the survival of restoration is durability of the material, the other is marginal and internal fit of restoration. Marginal accuracy of zirconia restorations has been investigated in various studies [19–21]. Only a few studies have investigated the effect of short-term sintering on the marginal accuracy of monolithic zirconia containing 3 mol% Y_2_O_3_ [22, 23]. The aim of this study was to investigate the effect of two different sintering times on the marginal and internal fit of three generations monolithic zirconia crowns. The null hypothesis was that there is no difference in the marginal and internal fit of restorations with different sintering times.

## Methods

**Fig. 1:**
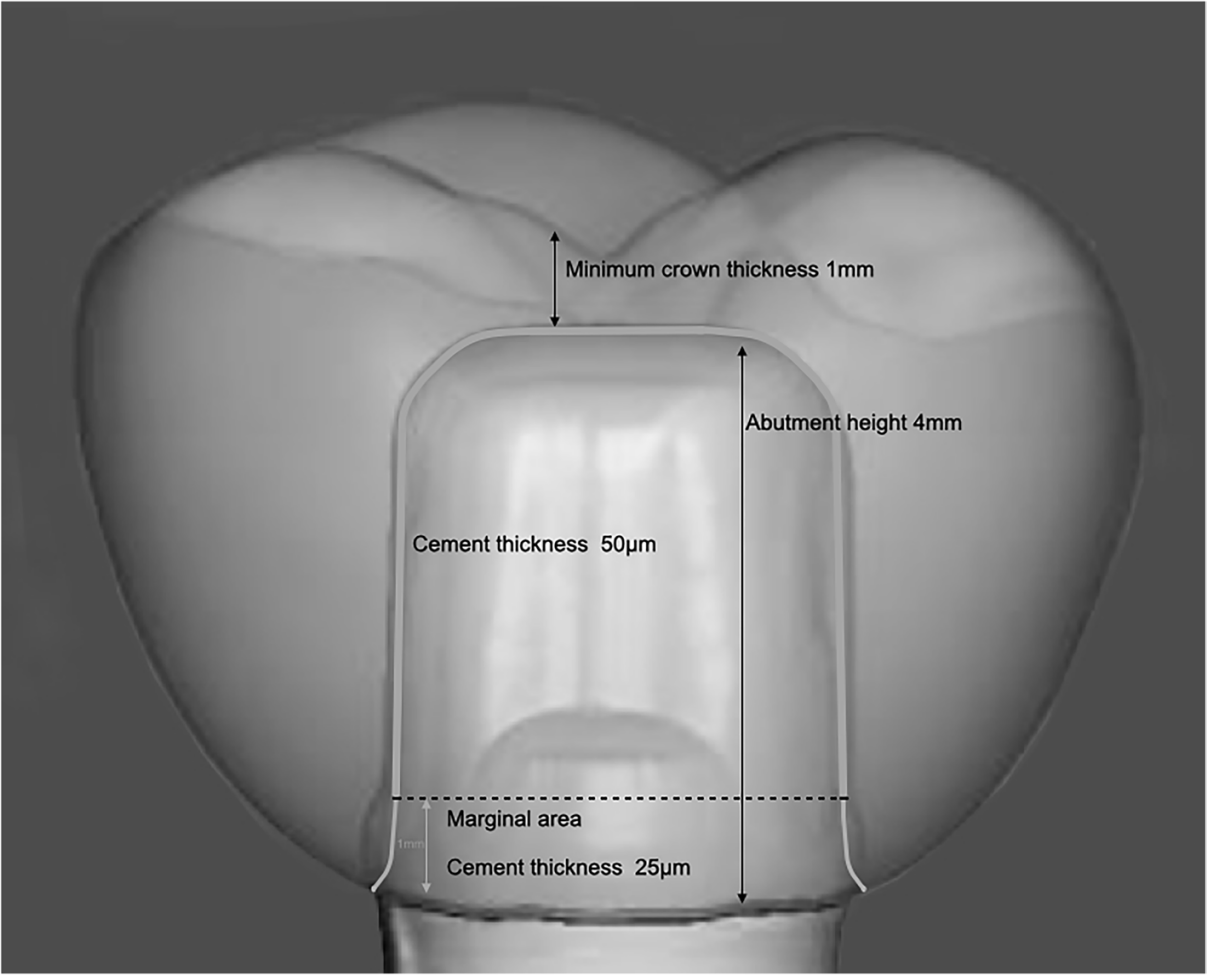
Designed crown on the prefabricated abutment

Sixty prefabricated abutments (solid abutments, 4 mm height; Straumann, Basel, Switzerland) were used to evaluate the marginal and internal accuracy of monolithic crowns (*n* = 10). A power analysis was conducted to determine the required specimen size for a two-way ANOVA design, and a specimen size of 10 in each group was found adequate to detect the effect size of 0.816 with a power of 80%. The abutments were connected to their corresponding implant analogs with screwdrivers and tightened with 35 Ncm torque with torque control devices. Implant analogs were embedded into epoxy resin (Morapox, Moravia) with a dental surveyor (Paraskop M, Bego GmbH). Each abutment was scanned with the Dental Wings 7series 3D Scanners (Dental Wings Inc., Montreal, Canada) after a scannable surface was achieved by powder spraying (Cerec Optispray, Sirona Dental Systems, Bensheim, Germany). A maxillary first molar tooth was designed with DWOS (Dental Wings Inc., Montreal, Canada) with a simulated cement space of 25 μ around the margins, and additional cement space of 50 μ from 1 mm above the finish lines of the abutment (Fig. 1) [24]. Groups were created according to the material composition (according to the manufacturer’s catalog): 3Y-TZP Generation 1 (alumina wt; ≤0.5%, cubic phase; < 15%); Lava™ Zirconia (3 M ESPE, Neuss, Germany), 3Y-TZP Generation 2 (alumina wt; ≤0.05%, cubic phase; < 15%); inCoris TZI (Dentsply Sirona, Bensheim, Germany) and 4Y-TZP (alumina wt; ≤0.05%, cubic phase; > 25%); Katana™ Zirconia STML (Kuraray Noritake Dental, Aichi, Japan).

The crowns in each zirconia generation group were assigned to the long-term and short-term sintering groups randomly by using opaque, sealed envelopes. The crowns were sintered according to the instructions using the same furnace (inFire HTC Speed, Sirona Dental Systems, Bensheim, Germany) by a single operator (Table 1).

**Table 1:** Performed sintering’s according to the protocols

Sintering protocol	Starting Degree	Heating rate	Sintering temperature	Holding time	Cooling down speed	Total sintering time (approximately)
**Long - term**	Room temperature (25 ± 2 °C)	10 °C / min	1500 °C	120 minutes	−10 °C / min	7 hours
**Short - term**		40 °C / min		45 minutes	−40 °C / min	2 hours

To analyze the fit of the crowns, marginal and internal gap were measured with the silicone replica technique [25]. A light-body type impression material (Vinylsiloxanether Identium Light; Kettenbach GmbH & Co KG) was applied to the inner surface of the crowns. Restorations were placed on the abutments with finger pressure for 5 s and then subjected to a 50 N load (5 kg) for 5 min by using a custom device which provides a constant load.

**Fig. 2:**
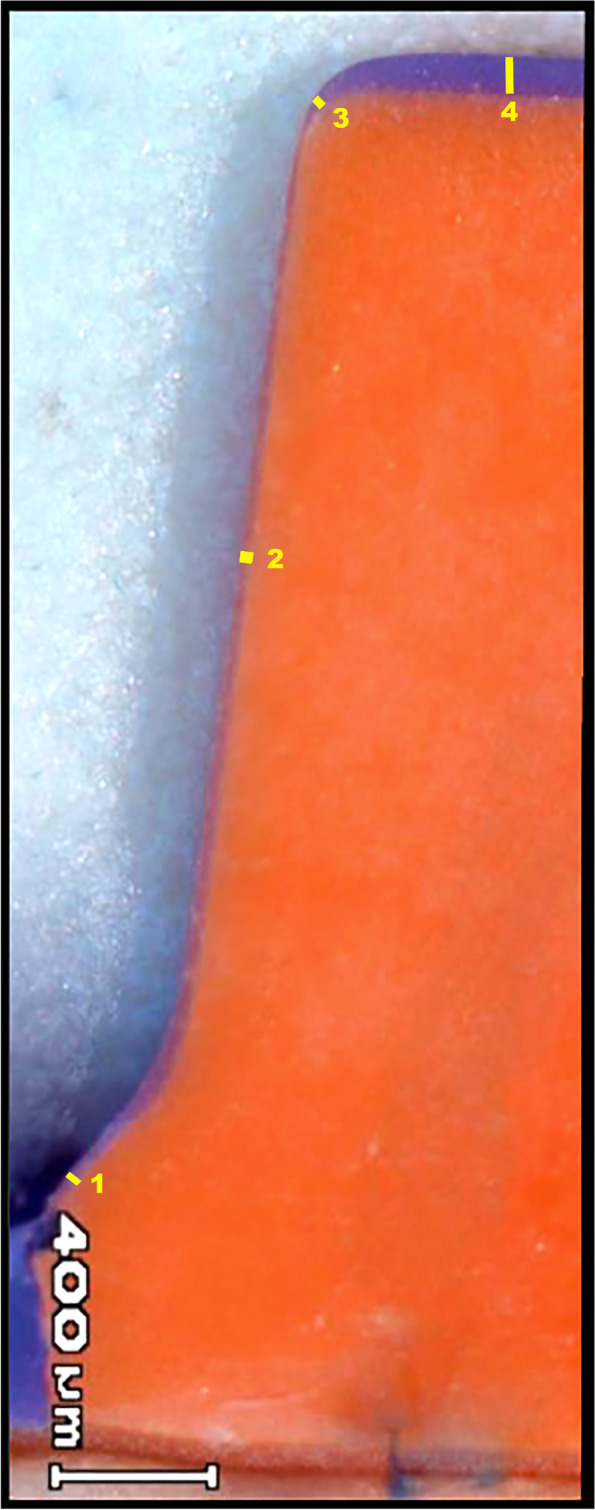
Silicone replica under light microscopy. Purple color indicates cement thickness. The yellow marked areas represent the measurement points for marginal and internal gap of the crowns. Marginal gap = (points 1), Internal gap = (point 2,3,4), Total cement thickness = (points 1, 2, 3, 4)

After the impression material polymerized, the restorations were removed from the abutment, leaving the impression material intact. To stabilize the thin elastomer layer, which represented the discrepancy between the abutment and the restoration, a light-body type of polyvinyl siloxane impression material (Elite HD+ Light Body Fast Set; Zhermack SpA) was applied. The silicone replicas were sectioned with a sharp blade in two directions, buccolingually and mesiodistally. Measurements were performed from each quarter part of the replica as follows: one marginal, two axial (mid-distance of the axial wall and the junction between the occlusal and axial area), and one occlusal (in the center of occlusal area). For each crown 16 reference (4 marginal and 12 internal) measurement points were recorded for total cement thickness calculation by one blinded observer. All the measurements were recorded by using a light microscope (SZ61/SZ51; Olympus Corp) at × 45 magnification (Fig. 2), and a digital measurement program (ImageJ; National Institutes of Health). The comparison between groups and sintering times were analyzed by using the two-way ANOVA. Levene’s test was used to assess the assumption of homogeneity of variances. Pairwise multiple comparisons were performed with the t-test. The significance level was set at 0.05.

## Results

In the present study totally 60 monolithic zirconia crowns were fabricated and 960 measurements were performed. The interaction effect was statistically significant for marginal gap and total cement thickness values (Table 2).

**Table 2:** Statistical significances of factors for mean marginal gap and total cement thickness values

Source	Significance^*^	
	Marginal	Total
Group	< 0.001	0.005
Timing	< 0.001	< 0.001
Interaction (Group ^*^ Timing)	**< 0.001**	**< 0.001**

^*^p-values were calculated from a two-way ANOVA model

**Fig. 3:**
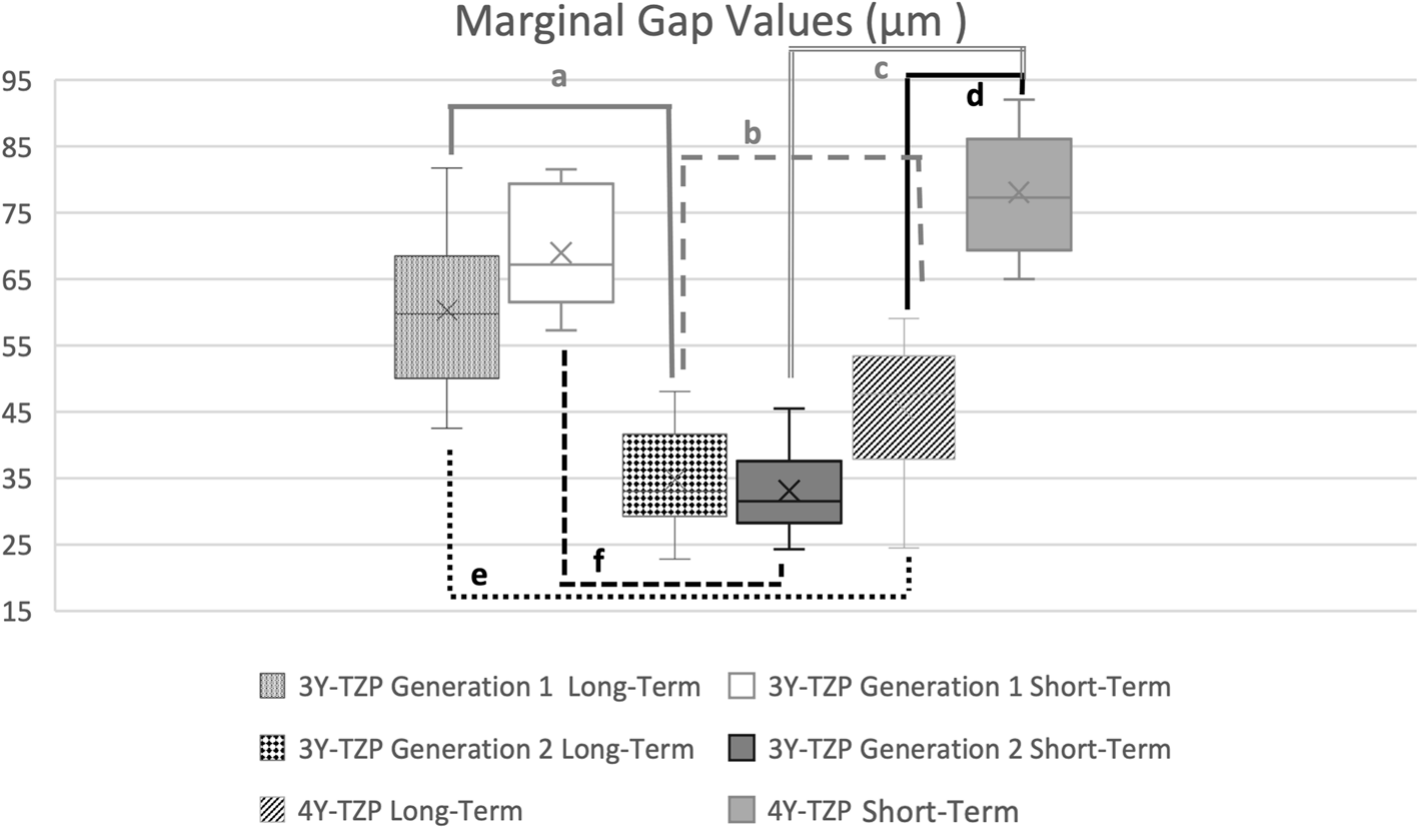
Mean marginal gap values (μm) of monolithic zirconia crowns. (**a ***p* < 0.0001, **b** p:0.016, **c ***p* < 0.0001, **d ***p* < 0.0001, **e** p:0.001, **f ***p* < 0.0001)

**Fig. 4:**
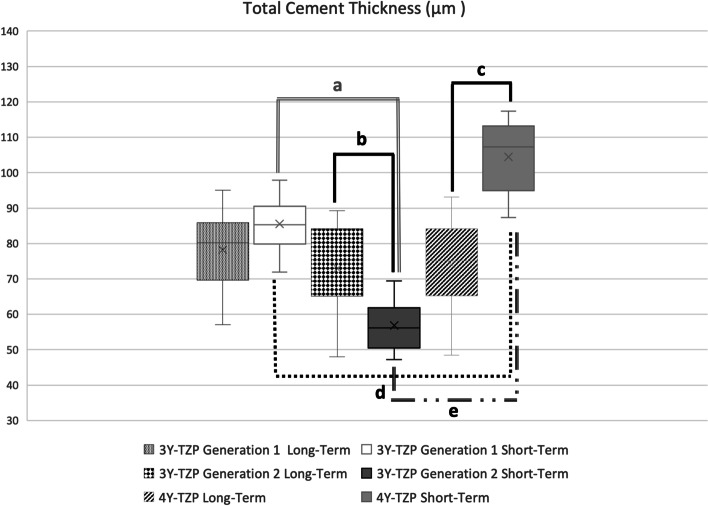
Mean total cement thickness values (μm) of monolithic zirconia crowns. (**a ***p* < 0.0001, **b ***p* = 0.0016, **c ***p* < 0.0001, **d ***p* < 0.0001, **e ***p* < 0.0001)

The mean marginal gap values were given in Fig. 3. The two-way ANOVA test revealed a statistical significance among the groups in both long term and short-term sintering protocols (*p* < 0.0001). When the impact of sintering time on the crowns was evaluated, statistical significance was detected only in 4Y-TZP group (*p* < 0.0001). The mean total cement thickness values (μm) were given in Fig. 4. While total cement thickness values were comparable in the long-term sintering protocol (*p* > 0.05), statistical significance was detected among the groups in the short-term sintering protocol (*p* < 0.0001). When the impact of sintering time on the crowns was evaluated, statistical significance was detected in 3Y-TZP Generation 2 (*p* = 0.0016) and 4Y-TZP (*p* < 0.0001).

## Discussion

The null hypothesis, that there is no difference in the accuracy of restorations with different sintering times, was partially rejected. The mean marginal gap values of 4Y-TZP were increased if the sintering time is shortened while 3Y-TZP groups were not affected by this situation.

A few studies focused on the effect of changes in the sintering time on the marginal fit of monolithic zirconia crowns [23, 26]. Khaledi et al. [23] reported that different sintering times does not alter the marginal fit of the zirconia copings fabricated from 3Y-TZP presintered blocks. The findings are in accordance with the present study for the 3Y-TZP group. On the other hand, in a previous study focused on 3Y-TZP monolithic crowns it was showed that the marginal gap values of short-term sintering (2 h, 55 minutes in total) groups were higher than the corresponding combinations of standard sintering (9 hours, 50 minutes in total) [26]. The differences between the findings of these studies may be due to the thickness of restorations.

4Y-TZP zirconia was launched in 2015 with the indication of full-contour restoration and framework material in the posterior region due to adequate mechanical strength with improved optical properties. The present study revealed a statistically significant increase in the marginal gap values in 4Y-TZP group for the short-term sintering protocol. The creep rate of polycrystalline ZrO2 with different composition may be more sensitive to creep and distortion during the sintering process [27]. Increasing yttria content influence the number of grain boundaries that controls the major physical properties such as light transmission and flexural strength [28]. Moreover, material composition of monolithic zirconia crowns affect internal fit, crown margin quality [29].

Zirconia restorations produced by soft machining of the presintered blocks are sintered at temperatures varying between 1350 and 1550 °C, depending on the manufacturer. In general, it can be stated that sintering temperatures of 1600 °C onwards lead to a decrease in flexural strength [14]. The sintering conditions have a strong impact on the mechanical properties of the definitive product [30]. Novel speed sintering protocols have been developed to meet the demand for chairside one-visit restorations [31]. Altering the sintering parameters was attempted to shorten the zirconia sintering process by inducing rapid heating rate and lowering the sintered-holding time [18].

For improved productivity, shortened dwell time is beneficial, but it may result in poor mechanical behavior compared with long-term sintering [18]. On the other hand, encouraging results of a previous study showed that the combination of high sintering temperature and short sintering time increases the flexural strength of zirconia [16]. Additionally, it was reported that biaxial flexural strength is not affected by altering the sintering conditions [15, 32]. More research is needed to assess the effect of sintering times and temperatures on the mechanical properties of zirconia prostheses.

Shrinkage occurring in presintered blocks during the sintering process has been studied previously [12, 33]. The milling machine software is programmed to compensate for the shrinkage percentage of the partially sintered zirconia blocks after sintering [30]. Rezende et al. [12] evaluated dimensional changes brought about by sintering of the Y-TZP blocks and reported a significant difference among the groups for the internal fit of copings. Additionally, the sintering shrinkage rate reported by the manufacturer was different from that obtained experimentally. Limited data are available to compare those with results of the present study. However, it is noteworthy that one of the two groups with a significant difference showed an increase in the gap values, whereas shrinkage occurred in the other group. This may be because the density difference of presintered zirconia blocks affects linear sintering shrinkage [33]. The shrinkage process is determined by numerous factors, such as the material itself, the compaction density, density distribution, and the parameters of the sintering process. The density distribution, known as a central characteristic in a blank, determines the local shrinkage and therefore the dimensional accuracy after final sintering [23].

Non-uniform sintering shrinkage might lead to a misfit of the restorations. The cement space has a significant effect on the marginal fit of monolithic zirconia crowns. Marginal discrepancy values increase when the cement space decreases. As the cement space was set at 50 μm using the software, the marginal discrepancy was 53 μm in this study. Conversely, when the cement space was set at 30 μm, the marginal discrepancy was 85 μm [24]. Larger internal misfit may also affect the success of ceramic crowns [34]. However, there is no consensus on the limit for clinically acceptable values of internal fit. The mean variation in the crowns tested in the present study is in accordance with that reported in previous studies [29, 35]. Additionally, marginal gap values of all groups were less than 120 μm. The widest marginal gap measured was 78 ± 9 μm, and the lowest was 33 ± 6 μm. Larger frameworks might result in a higher misfit of the prosthesis [36].

The silicone replica technique was chosen to analyze the fit of crowns. Sixteen assessment points for each crown were determined to evaluate the fit. The silicone replica technique is a nondestructive, less costly, and less time-consuming method for evaluating the adaptation of restorations. The evaluation of internal adaptations at different regions of the restorations is possible using this technique [25]. Although this technique is prone to inconsistencies due to manual discrepancies and errors, the limitation of the technique is consistent for all crowns. Further studies are required to assess the effect of sintering conditions on the accuracy of new generations of monolithic zirconia restorations.

## Conclusions

Within the limitations of the present study:

1. Altering the sintering time had no effect on the marginal fit of the crowns manufactured from 3Y-TZP groups.
2. Shortening the sintering time for 4Y-TZP monolithic crowns resulted in significantly larger gap values.

## Data Availability

The datasets used and/or analysed during the current study are available from the corresponding author on reasonable request.

## Abbreviations

3Y-TZP: Three mol% yttria-stabilized tetragonal zirconia polycrystal
4Y-TZP: Four mol% yttria-stabilized tetragonal zirconia polycrystal
